# Identity Matters: Validation of the Professional Identification Scale in a Sample of Teachers in South Africa During the COVID-19 Pandemic

**DOI:** 10.1007/s43076-022-00225-z

**Published:** 2022-08-10

**Authors:** Tyrone B. Pretorius, Anita Padmanabhanunni, Serena Ann Isaacs

**Affiliations:** Department of Psychology, University of the Western Cape, Robert-Sobukwe Road, Private Bag X17, BellvilleCape-Town, 7535 South Africa

**Keywords:** Professional identification, Rasch analysis, Mokken analysis, Classical test theory, Reliability, Validity

## Abstract

Professional identity has been linked to organizational outcomes such as job performance and commitment, as well as health and well-being indices such as burnout and depression. Professional identity is a powerful mechanism that can be affected by social and environmental factors. It is therefore important to establish a reliable and valid measurement of professional identity that is useful in different contexts. The current study examines the psychometric properties of Brown’s Professional Identification Scale (PIS) using three different but complementary approaches: classical test theory, Mokken analysis, and Rasch analysis. The study participants (*N* = 355), school teachers recruited from all over South Africa, completed the PIS, the Satisfaction with Life Scale, and the Teacher Satisfaction Scale. The reliability and validity of a reduced 8-item version of the PIS was confirmed. Mokken and Rasch analyses indicated that the scale consists of more than one dimension, and classical test theory (exploratory and confirmatory factor analysis) supported a two-factor structure. Ancillary bifactor indices indicated that professional pride and professional discontent explained a sufficient amount of the variance over and above that explained by the professional identity as a total scale. Overall, the findings support an 8-item PIS for use in a South African teacher population.

The COVID-19 pandemic represents a significant public health crisis across the world. As of 19 January 2022, it has resulted in more than 300 million cases and 5.5 million deaths. In South Africa, cases have passed the 3 million mark and deaths have exceeded 90,000 (Worldometer, 2022).

In March 2020, at the start of the pandemic in South Africa, the government swiftly introduced emergency protocols with five levels of lockdown regulations, of which Level 5 is the most drastic. Many sectors in society, such as retail and hospitality, were adversely affected by these protocols; however, the education sector was arguably one of the worst affected. All schools were closed during lockdown Level 5 and reopened under lockdown Level 4 (South African Government, 2022). Lockdown Level 5 restrictions, as well as the less severe Level 4 restrictions enforced during the second wave (December 2020–January 2021) of the virus in South Africa, significantly impacted the academic program, the learning and teaching of learners, and extensions of the 2020–2021 academic year. As in the rest of the world, schools and universities in South Africa had to quickly migrate to remote and online modes of teaching and learning. This shift severely affected learning, because a large number of South African students had no access to data or Internet or lacked the necessary technology skills. The Department of Basic Education eventually introduced a rotational system, under which learners could attend school in person on a daily or weekly rotation. The impact of the disruptions in learning and teaching due to the COVID-19 pandemic and resulting restrictions has been challenging, and this impact persists among learners and teachers alike.

UNICEF has described the pandemic’s effect on education as “devastating” and maintains that South African school children have lost 54% of learning time. Additionally, nearly 700,000 students have dropped out of school (UNICEF, 2021). Studies have reported that children have experienced higher levels of psychological distress during the pandemic than before. For example, Qin and colleagues (2021) found that self-reported psychological distress among children during the COVID-19 pandemic was higher than that reported in a similar study from 2017. Tang and colleagues (2021) found that 24.9% of pupils in their study sample reported symptoms of anxiety, 19.7% reported symptoms of depression, and 11.5% met thresholds of depression, anxiety, and stress.

The impact of COVID-19 on teachers has been equally severe, and many studies across the world have painted a bleak picture of the pandemic’s negative psychological impact on school teachers. Ozamiz-Etxebarria and colleagues (2021) found that 50.5% of their study sample of teachers in Spain reported suffering from stress, 49.5% reported suffering from anxiety, and 32.2% reported suffering from depression. Similarly, a study in Greece found that 34% of teachers were very anxious during the pandemic and 8% exhibited severe depressive symptoms (Stachteas & Stachteas, 2020). A Polish study found that 44.14% of teachers reported depressive symptomatology, 46.89% reported anxiety, and 45.52 reported stress. Levels of depression, anxiety, and stress among the study sample ranged from mild to very severe (Jakubowski & Sitko-Dominik, 2021). In addition to the negative psychological effects associated with COVID-19 and its impact on teachers’ well-being, it is important to consider how the pandemic’s effects have impacted teachers’ view of themselves as teachers, also known as their professional identity.

Professional identity refers to the extent of one’s identification with teaching as a profession and includes the beliefs, values, and commitment an individual has toward the teaching profession (Richardson & Watt, 2018). Professional identification is an important mechanism that affects workplace performance, work-related behavior, and mental health. Several scholars (e.g., Beijaard et al., 2004; Karaolis & Philippou, 2019) have acknowledged that professional identity is neither stable nor fixed. It is also ever evolving and can therefore be impacted by contextual, cultural, and environmental factors (Rosdi et al., 2020).

In organizational psychology, professional identification has been identified as a predictor of job satisfaction (e.g., Martin, 2020) organizational commitment (e.g., Afshari et al., 2019) and turnover intention (e.g., C. Wang et al., 2020). It has also been negatively associated with burnout (e.g., Correia & Almeida, 2020) and mental health (e.g., L. Wang et al., 2019). Additionally, professional identification has been identified as a protective factor that can mediate (e.g., Padmanabhanunni & Pretorius, 2021) or moderate (e.g., Asplund, 2020) the impact of adverse factors. For example, the findings of a qualitative study investigating teachers’ sense of professional identity in Ireland illustrate that teachers felt “lost and confused” and isolated at the beginning of the COVID-19 pandemic, in contrast to the sense of their transformed teacher identity later in the pandemic (Mellon, 2021). Mellon (2021) describes teacher identity as a “powerful resource” that is useful in teaching practices, connecting with learners, and providing teachers with a positive sense of hope for their future within the educational context. In a meta-analysis of three types of identification targets—team, organization, and profession—Greco and colleagues (2021) found that team and professional identity explained more variance in job performance than organizational identity. Organizational identity and professional identity explained more of the variance in psychological well-being than team identity. Professional identity is an important concept and process that can affect personal well-being and teaching practices (Kavrayici, 2020) and ultimately deepen one’s sense of commitment to the education profession (Mellon, 2021). Similarly, Thomas & Beauchamp (2007) emphasize the importance of raising awareness of professional identity development among current and future teachers.

The significance of professional identity necessitates the development and validation of measures that are applicable in different contexts. Measures that demonstrate cross-cultural applicability facilitate the search for psychological universals and identification of cultural differences. The current study was undertaken in South Africa where teacher identity has been shaped by distinctive factors including the legacy of Apartheid and continued social inequality and disparities in access to resources (Davids, 2018). Historically, teachers taught in schools that matched their racial identity. With the transition to democracy in 1994, the South African government introduced “Norms and Standards for Educators” (Department of Education, 1997) which mandates teacher training institutions to not only prepare teachers to service the formal basic education system but to be agents of change within communities and to promote the ideals of democratic citizenship (Davids, 2018). Hence, teachers in the country have to balance their professional identity, which is often influenced by policy, with their personal identity as a teacher which is often linked to their values and life experiences (Davids, 2018). Although teaching remains an undervalued profession in the country, personal and professional identification with the profession has a significant bearing on teacher retention (Du Plessis & Mestry, 2019). A significant portion of schools in South Africa are located in rural settings and are characterized by poor infrastructure, lack of basic necessities such as running water, and limited information communication technology (Du Plessis & Mestry, 2019). Teachers in rural communities are often expected to take on multiple duties without the commensurate financial compensation. Those that remain in these settings do so from a vested interested in promoting access to education among students and from a desire to uplift these communities (Du Plessis & Mestry, 2019).

Given the nature of professional identity and its sensitivity to change due to external factors (Mellon, 2021), it is necessary to have tools and measures that can identify professional identity to strengthen professional identification processes. Brown and colleagues (1986) developed a 10-item scale of professional identification, based on social identity theory, that relies on three aspects: awareness of group membership, evaluation, and affect. The authors only reported the reliability of the scale and offered a cursory examination of validity.

A review of the existing literature identified that there has not been a comprehensive examination of the reliability and validity of the scale or its application in South Africa. Hence, the current study examines the applicability of the Professional Identification Scale in South Africa. Specifically, the study focuses on the psychometric properties of the Professional Identification Scale from three different perspectives: classical test theory (CTT), Rasch analysis, and Mokken analysis.

Most measurement tools in the social sciences have been developed using CTT. It is a common approach in validation studies that is used to analyze latent variables and reliability of a given measure. As functions of item response theory, Mokken and Rasch analyses lead to similar results. Mokken scale analysis is a nonparametric approach best conducted in studies in which latent variables are measured with a small number of items (van Schuur, 2003). Rasch analysis, a popular model of item response theory, takes a more stringent parametric approach to data analysis (Engelhard, 2008). The use of CTT in combination with Rasch and Mokken analyses is an effective approach to enhance validation efforts (Cappelleri et al., 2014) and is therefore considered advantageous in cross-cultural research.

## Method

### Participants

The study participants were 355 school teachers recruited from all over South Africa. In 2019, South Africa reportedly had about 444,000 teachers (Galal, 2021); therefore, the sample size of 355 represents a margin of error of 5.09% with a 95% confidence interval. The majority of the sample were from the Western Cape Province (82.3%), women (76.6%), and residents of an urban area (61.7%). The mean age of the sample was 41.89 years (SD = 12.42).

### Instruments

Participants completed a brief demographic questionnaire, as well as the following measures: the Professional Identity Scale (PIS: Brown et al., 1986), the Teaching Satisfaction Scale (TSS: Ho & Au, 2006), and the Satisfaction with Life Scale (SWLS: Diener et al., 1985). All participants completed the survey in English. The PIS consists of 10 items and measures the extent to which teachers identify with the teaching profession. It is scored on a 5-point Likert scale that ranges from Never (1) to Very Often (5). A sample item of the PIS is “I am a person who sees myself as belonging to the teaching profession.” In the first application of the scale, the authors reported an alpha coefficient of 0.71, and a significant difference between respondents who made positive and negative comments during an interview was taken as evidence of the scale’s validity. Subsequent applications of the PIS have generally reported alpha coefficients greater than 0.70 (e.g., Lu et al., 2007; Sun et al., 2020).

The TSS is a 5-item measure of the job satisfaction of teachers, and it is scored on a 5-point Likert scale that ranges from Strongly Disagree (1) to Strongly Agree (5). A sample item of the TSS is “My conditions of being a teacher are excellent.” In the original development study, the authors reported an alpha coefficient of 0.77. Convergent validity was established through positive correlations between teaching satisfaction and two different measures of job satisfaction. Negative correlations between teaching satisfaction and psychological distress, as well as teacher stress, provided evidence of criterion-related validity. Other studies have also reported reliability coefficients greater than 0.70 (e.g., Han et al., 2021; Nalipay et al., 2019).

The SWLS scale is the most widely used measure of life satisfaction. It consists of 5 items scored on a 7-point scale that ranges from Strongly Disagree (1) to Strongly Agree (7). A sample item of the SWLS is “In most ways my life is close to my ideal.” In the original study, the authors reported an alpha coefficient of 0.87. In terms of validity, the SWSLS has been significantly correlated with other measures of well-being. The SWLS scale has been used in various countries (e.g., Germany: Hinz et al., 2018; France: Bacro et al., 2020), and estimates of the scale’s internal consistency have typically been greater than 0.75.

### Procedure

Google Forms was used to construct an electronic version of the instruments. With the permission of administrators of teacher Facebook groups, the link was posted on Facebook and teachers were invited to participate. Some teachers also requested that the link be emailed to them.

### Ethics

The Humanities and Social Sciences Ethics Committee of the University of XXX provided ethical approval to perform the study (ethics reference number: HS21/3/8). The participants were assured of the voluntary nature of participation and anonymity. They were required to provide informed consent on the first page of the survey before being allowed to proceed. As the questionnaires had the potential to cause distress, the participants were also provided with the contact details of counseling services that can be utilized free of charge.

### Data Analysis

CTT analyses were conducted using IBM SPSS Statistics version 27 for Windows (IBM Corp., Armonk, NY, USA), and CFA were performed using IBM SPSS Amos version 27 (IBM Corp.). Ancillary bifactor indices, which included explained common variance (ECV), Omega hierarchical (OmegaH), and the percentage of uncontaminated correlations (PUC), were calculated using the Bifactor Indices Calculator (Dueber, 2017). Rasch analyses were conducted using Winsteps version 5.1.4 (Linacre, 2021), and Mokken analyses were performed with R using the “Mokken” package (van der Ark, 2012).

#### Reliability

Indices of reliability included Cronbach’s alpha (α) and composite reliability (CR) from the perspective of CTT and Mokken scale reliability (MS_rho_). For Cronbach’s alpha, *CR*, and *R*_*ho*_, the conventional cut-off of 0.70 is considered acceptable reliability (Taber, 2018).

#### Validity

Construct validity was examined through model fit indices in CFA, item-total correlation, and the item and person separation and fit indices obtained in Rasch analysis. A good model fit in CFA provides evidence that the items of the latent construct sufficiently reflect the latent construct. As suggested by Kline (2005), the model fit indices used included chi-square (χ^2^_:_ best if *p* > 0.05), root mean square error of approximation (RMSEA: best if < 0.08), comparative fit index (CFI: best if > 0.90), goodness of fit index (GFI: best if > 0.95), and Tucker–Lewis index (TLI: best > 0.90). Additionally, Akaike’s information criterion (AIC, lower values indicate better fit), which is generally used for model comparisons, was included.

The item-total correlations also provide a measure of the extent to which each item contributes to the latent construct. According to Hajjar (2018), item-total correlations of > 0.50 provide further evidence of construct validity, as do item and person separation indices from the Rasch analysis. Linacre (2021) suggests that person separation index > 2 and person separation reliability > 0.80 provide evidence that the scale is sensitive enough to distinguish between high and low performers. On the other hand, item separation index > 3 and item separation reliability > 0.80 provide evidence that an item difficulty hierarchy exists. With respect to item and person separation reliability, it has also been proposed that < 0.67 is poor, 0.67–0.80 is fair, 0.81–0.90 is good, and > 0.91 is very good (Mohamad et al., 2015). Rasch analysis also provides two fit statistics, infit and outfit mean square (MnSq), which are used to measure how well the data fit the model. Mean square values between 0.5 and 1.5 are considered ideal (Linacre, 2021).

In Mokken analysis, monotonicity and invariant item ordering (IIO) also provide evidence of construct validity. Monotonicity refers to the probability that the likelihood of a particular response level is a monotonically nondecreasing function of the latent construct, whereas IIO describes the extent to which items have the same order irrespective of the level of the latent variable (Sijtsma & van der Ark, 2017). Mokken analyses provide a Crit value to determine whether there are violations of these two assumptions. Generally, Crit values > 80 indicate serious violations, and Crit values between 40 and 80 indicate minor violations that are acceptable (Sijtsma & van der Ark, 2017). The presence of violations indicates that items do not distinguish well between those with high and low levels of professional identification (monotonicity) and that respondents with the same level of professional identification might respond in significantly different ways to the items with violations (IIO). Differential item functioning (*DIF*) across gender and rural/urban groups obtained through Rasch analysis provides further evidence of validity. Measurement invariance across groups is demonstrated if *DIF* < 0.50.

Other aspects of construct validity that were evaluated include convergent validity, discriminant validity, and concurrent validity. Convergent validity was examined through factor loadings, average variance extracted (AVE), and CR. All factor loadings should be significant and AVE should be > 0.50 and lower than CR (Almén et al., 2018) to demonstrate convergent validity. To demonstrate discriminant validity, the latent construct (professional identification) should explain more of the variance in the items than the variance that the construct shares with other constructs. In this regard, AVE should be greater than the maximum shared variance (MSV) or average shared variance (ASV, Almén et al., 2018).

Concurrent validity is evidenced by the relationship between the construct and other variables it is expected to correlate with. Several studies have reported a link between professional identity and job satisfaction (e.g., Chen et al., 2020; Padmanabhanunni & Pretorius, 2021). Additionally, although no studies were found to have examined the relationship between professional identity and life satisfaction, several studies have reported a strong relationship between job satisfaction and life satisfaction (e.g., Bernarto et al., 2020; Bialowolski & Weziak-Bialowolska, 2021). A significant relationship between professional identity and job satisfaction, as well as life satisfaction, would therefore support concurrent validity.

#### Dimensionality

Evidence for dimensionality was obtained through CTT, Mokken, and Rasch analyses. In Mokken analysis, an automated algorithm called the automated item selection procedure (AISP) indicates whether the scale is unidimensional or multidimensional. It also provides a scalability coefficient for each item (*H*_*i*_) and for the total scale (*H*). The following guidelines are suggested for evaluating *H*-coefficients: *H* ≥ 0.50 indicates a strong scale, 0.40 ≤ *H* < 0.50 indicates a medium scale, and 0.30 ≤ *H* < 0.40 indicates a weak scale (Sijtsma & van der Ark, 2017).

In Rasch analysis, dimensionality is examined through a principal component analysis (PCA) of residuals. If the eigenvalue associated with a possible second dimension (referred to as the “first contrast”) is greater than 2, it is likely that the instrument is multidimensional (Linacre, 2021). In addition to the fit indices provided by CFA, ancillary bifactor indices provide an indication of dimensionality. ECV measures the amount of variance accounted for by the general factor (or total scale), whereas ECV_S_ refers to the amount of variance explained by the specific factors (or subscales). If ECV > 0.80, the scale is regarded as essentially unidimensional. OmegaH measures the proportion of variance in scale scores that are due to individual differences in the general factor; an OmegaH of > 0.80 indicates that the scale is unidimensional (Rodriguez et al., 2016). PUC is a measure of the number of unique correlations among items that are accounted for by the general factor. These three indices should not be examined in isolation; taken together, PUC < 0.80, ECV > 0.60, and OmegaH > 0.70 would indicate the presence of some multidimensionality that is not strong enough to rule out the interpretation of the instrument as essentially unidimensional.

Lastly, we report on the test information function of the scale as obtained through Rasch analysis. The test information function (TIF) is an aggregation of the plots of the individual items which are referred to as item information curves. The TIF shows the degree of precision at different values of the latent variable, namely professional identity.

## Results

The descriptive statistics reliabilities (coefficient alpha) and intercorrelations between the study variables are reported in Table 1. The mean score for professional identification (*M* = 40.1, SD = 6.9) was similar to that reported in the original study (Brown et al., 1986: *M* = 41.0, SD = 5.4), as well as a study by Sun and colleagues (2020: *M* = 38.74, SD = 6.54). The theoretical range of scores is 10–50; thus, a mean of 40.1 indicates that the sample reported a very strong sense of professional identity. This finding is confirmed by the fact that 53% of the sample scored above the mean.

**Table 1:** Intercorrelations between variables, descriptive statistics, and reliabilities

	1	2	3
1. Professional identification	—		
2. Teaching satisfaction	.58^***^	—	
3. Life satisfaction	.32***	.46***	—
Mean	40.1	17.3	21.9
*SD*	6.9	4.7	7.3
Alpha	.85	.87	.90

^***^ *p* < .001

The internal consistency of all the scales can be considered satisfactory (α = 0.85–0.90). The internal consistency of the PIS compares favorably to previously reported findings (Brown et al., 1986: α = 0.71; Lu et al., 2007: α = 0.82; Sun et al., 2020: α = 0.82). In terms of the intercorrelation between variables, professional identity was positively related to teaching satisfaction (*r*_353_ = 0.58, *p* < 0.001) and life satisfaction (*r*_353_ = 0.32, *p* < 0.001) which provides support for concurrent validity.

The inter-item correlations and the CTT, Rasch, and Mokken indices at the item level are reported in Table 2. Except for items 7 and 10 (“I am a person who criticizes the teaching profession” and “I am a person who is held back by the teaching profession”), all item-total correlations were above 0.50. The infit and outfit MnSq values were acceptable and ranged between 0.7 and 1.4 (except for item 7, which exceeded 1.5). Items 7 and 10 also had the lowest scalability coefficients (*H*_*i*_ = 0.22 and 0.33, respectively). All other scalability coefficients for the individual items were acceptable and indicated that these items contributed to the measurement of the latent variable, professional identification. One violation of monotonicity (item 7) and 7 violations of IIO were identified, but all Crit values were below the suggested level of 80, which indicates no serious violations of monotonicity or IIO. The ten items of the PIS also reflected measurement invariance across gender (DIF = 0.08 to 0.44) and rural/urban groups (DIF = 0.00 to 0.39), as all items had a DIF < 0.50.

**Table 2:** Inter-item correlations and indices for the professional identification scale at the item level

Item	1	2	3	4	5	6	7	8	9	10
1. Teaching profession important	—									
2. Annoyed to say member	.32	—								
3. Identifies with profession	.63	.33	—							
4. Tries to hide belonging	.26	.50	.26	—						
5. Feels strong ties to profession	.57	.38	.69	.28	—					
6. Glad to belong to profession	.57	.40	.62	.28	.76	—				
7. Criticizes profession	.09	.25	.05	.31	.10	.12	—			
8. Sees self belonging to profession	.58	.39	.63	.26	.70	.76	.18	—		
9. Makes excuses for belonging	.27	.41	.26	.63	.29	.30	.26	.26	—	
10. Feels held back by profession	.17	.42	.19	.32	.29	.33	.32	.31	.34	—
Mean	4.5	3.9	4.2	4.2	4.0	4.0	3.5	4.1	4.2	3.6
*SD*	0.8	1.2	.9	1.1	1.0	1.1	1.2	1.0	1.1	1.3
Item-total correlations	.56	.58	.59	.53	.67	.69	.29	.67	.52	.46
Infit MnSq (Rasch)	0.8	1.0	0.8	1.3	0.7	0.7	1.6	0.7	1.2	1.3
Outfit MnSq (Rasch)	0.8	1.0	0.9	1.0	0.7	0.7	1.8	0.9	1.1	1.4
H_i_ (Mokken) ^a^	.42	.41	.42	.39	.46	.47	.22	.46	.39	.33
SE of H (Mokken)	.03	.03	.03	.03	.03	.04	.03	.03	.03	.03
*Crit* value for monotonicity	0	0	0	0	0	0	19	0	0	0
*Crit* value for IIO	0	0	50	39	33	33	40	0	36	34
DIF across gender	.08	.03	.24	− .43	− .33	.43	− .44	.44	− .33	− .16
DIF across rural/urban	− .11	.09	.00	.42	− .19	− .39	.29	− .11	.04	− .15

^a^ Scalability coefficient of individual items

The Rasch, Mokken, and CTT indices for two versions of the PIS are reported in Table 3. The first version is the 10-item scale, and the second is an 8-item scale from which items 7 and 10—which were identified as not contributing to the measurement of professional identity—were removed. In general, the indices for the 8-item scale were marginally better in some instances and exceeded the suggested cut-off points in others. Both versions of the PIS demonstrated acceptable reliability (*α* = 0.85 and 0.86, CR = 0.89 and 0.90, *R*_*ho*_ = 0.90 and 0.87). However, the 10-item version (AVE = 0.45) failed to meet the minimum acceptable AVE threshold of > 0.50, while the 8-item version exceeded this threshold (AVE = 0.53). The 8-item version also had a smaller SEM (SEM = 1.87) than the 10-item version (SEM = 2.35). In terms of the scalability coefficient, the 8-item version demonstrated a stronger Mokken scale (*H* = 0.48) than the 10-item version (*H* = 0.39). Both versions had very good item separation reliabilities (0.93 and 0.95) and item separation indices (3.75 and 4.35). However, the person separation reliability and index for both versions may be considered fair rather than good (Mohamad et al., 2015). For both versions of the scale, AVE (0.45 and 0.53) was greater than MSV (0.34) and ASV (0.22). This finding indicates that the latent variable is better explained by its observed items than its correlation with other variables. In terms of dimensionality, the AISP in Mokken analysis identified item 7 as a misfitting item, and a PCA of residuals in Rasch analysis indicated the existence of a possible second factor with an eigenvalue greater than 2.

**Table 3:** Indices for the professional identification scale at the scale level

Index	One-factor model	One-factor (8 items)	Suggested cutoff
Cronbach’s α	.845	.861	> .7
Composite reliability	.885	.896	> .7
Average variance extracted (AVE)	.450	.528	> .5
Maximum shared variance (MSV)	.336	.336	AVE > MSV
Average shared variance (ASV)	.217	.217	AVE > ASV
Standard error of measurement	2.35	1.87	Small values
Item separation reliability (Rasch)	.93	.95	> .8
Item separation index (Rasch)	3.75	4.35	> 3
Person separation reliability (Rasch)	.72	.69	> .8
Person separation index (Rasch)	1.59	1.48	> 2
Unexplained variance in the first contrast (Rasch) ^a^	2.96	2.69	< 2
Scale H (Mokken)	.389	.475	> .50
Mokken scale reliability (MS_Rho_)	.896	.869	> .70

^a^ Eigenvalue

The results of EFA (principal components with varimax rotation) are reported in Table 4. The factor analysis extracted two factors, which explained 45% and 16% of the variance, respectively. All the positive items loaded on one factor, and all the negative items loaded on the second factor. These two factors were consequently labelled “professional pride” and “professional discontent,” each of which consisted of five items. Cronbach’s alpha for the two factors were 0.90 for pride and 0.75 for discontent. The deletion of items 7 and 10, as indicated by the item analysis, reduced the discontent subscale to 3 items with a Cronbach’s alpha of 0.76. Professional pride was positively related to teaching satisfaction (*r*_353_ = 0.57, *p* < 0.001) and life satisfaction (*r*_353_ = 0.35, *p* < 0.001), whereas professional discontent was negatively related to teaching satisfaction (*r*_353_ =  − 0.37, *p* < 0.001) and life satisfaction (*r*_353_ =  − 0.15, *p* < 0.01).

**Table 4:** Results of an exploratory factor analysis of the professional identification scale

Items	Factor loadings	
	1	2
**Factor 1: professional affiliation and pride**		
Teaching profession important	**.76**	.13
Identifies with profession	**.84**	.11
Feels strong ties to profession	**.86**	.18
Glad to belong to profession	**.85**	.21
Sees self belonging to profession	**.84**	.20
**Factor 2: professional discontent**		
Annoyed to say member	.34	**.65**
Tries to hide belonging	.16	**.79**
Criticizes profession	-.04	**.62**
Makes excuses for belonging	.18	**.74**
Feels held back by profession	.20	**.63**

The extraction method was a principal components analysis with varimax rotation

**Fig. 1:**
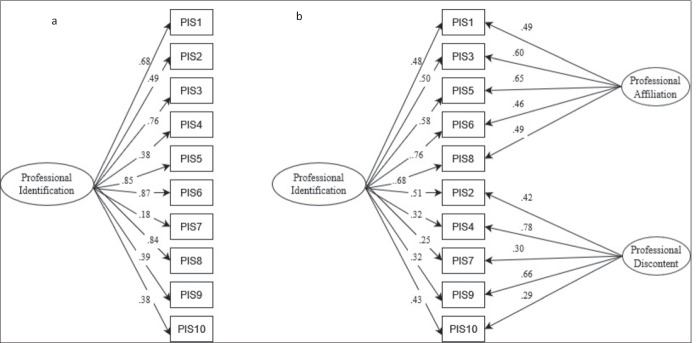
One-factor and bifactor models of the professional identification scale. Rectangles are measured variables. Ellipses are latent variables. Regression weights are standardized. **a** One-factor model, **b** bifactor model. PIS, Professional Identification Scale. All regression weights are significant at *p* < .05

CFA was used to test four conceptualizations of the PIS—two one-factor models consisting of 10 and 8 items, respectively, and two bifactor models based on the 10- and 8-item versions of the PIS. The one-factor and bifactor models based on 10 items are shown in Fig. 1.

The fit and bifactor indices are reported in Table 5. All four models demonstrated satisfactory fit indices; however, the one-factor model with 8 items (*χ*^2^_30_ = 68.23, GFI = 0.96, TLI = 0.97, CFI = 0.98, RMSEA = 0.06) and the bifactor model with 8 items (*χ*^2^_27_ = 65.28, GFI = 0.96, TLI = 0.96, CFI = 0.98, RMSEA = 0.06) had marginally better fit indices than the one-factor model with 10 items (*χ*^2^_44_ = 126.37, GFI = 0.94, TLI = 0.94, CFI = 0.96, RMSEA = 0.07) or the bifactor model with 10 items (*χ*^2^_44_ = 106.39, GFI = 0.95, TLI = 0.95, CFI = 0.97, RMSEA = 0.06). AIC also indicated that the 8-item one-factor and bifactor models were superior to the 10-item models. All the factor loadings were significant (*p* < 0.05). The ancillary bifactor indices indicated that for both bifactor models, the general factor explained only 47% of the variance (ECV = 0.47), and the two specific factors explained 53% of the variance (ECV_s1_ = 0.26 and 0.25, respectively; ECV_s2_ = 0.27 and 0.28, respectively). The joint consideration of ECV with OmegaH and PUC indicates that the PIS should be regarded as multidimensional (PUC < 0.80, ECV < 0.60, OmegaH < 0.70).

**Table 5:** Goodness of fit and bifactor indices for four models of the professional identification scale

Index	Best fit indicator	One-factor model	One-factor (8 items)	Bifactor model	Bifactor (8 items)
Goodness of fit					
χ^2^ (df)		126.37 (44)	68.23 (30)	106.39 (44)	65.28 (27)
*p*-value	*p* > .05	*p* < .001	*p* < .001	*p* < .001	*p* < .001
*GFI*	> .95	.94	.96	.95	.96
*TLI*	> .90	.94	.97	.95	.96
*CFI*	> .90	.96	.98	.97	.98
*RMSEA*	< .08	.07	.06	.06	.06
*AIC*	Lower levels	194.37	118.23	174.40	121.28
Bifactor					
*ECV*	—	—	—	.47	.47
*ECV*_*S1*_	—	—	—	.26	.25
*ECV*_*S2*_	—	—	—	.27	.28
*OmegaH*	—	—	—	.56	.55
*PUC*	—	—	—	.47	.53

χ^2^, chi-square statistic; *GFI*, goodness of fit index; *TLI*, Tucker–Lewis index; *CFI*, comparative fit index; *RMSEA*, root-mean-square error of approximation; *SRMR*, standardized root-mean-square residual; *AIC*, Akaike information criterion; *ECV*, explained common variance of general factor; *ECV*_*S1*_, explained common variance of specific factor 1; *ECV*_*S2*_, explained common variance of specific factor 2; *PUC*, percentage of uncontaminated correlations

**Fig. 2:**
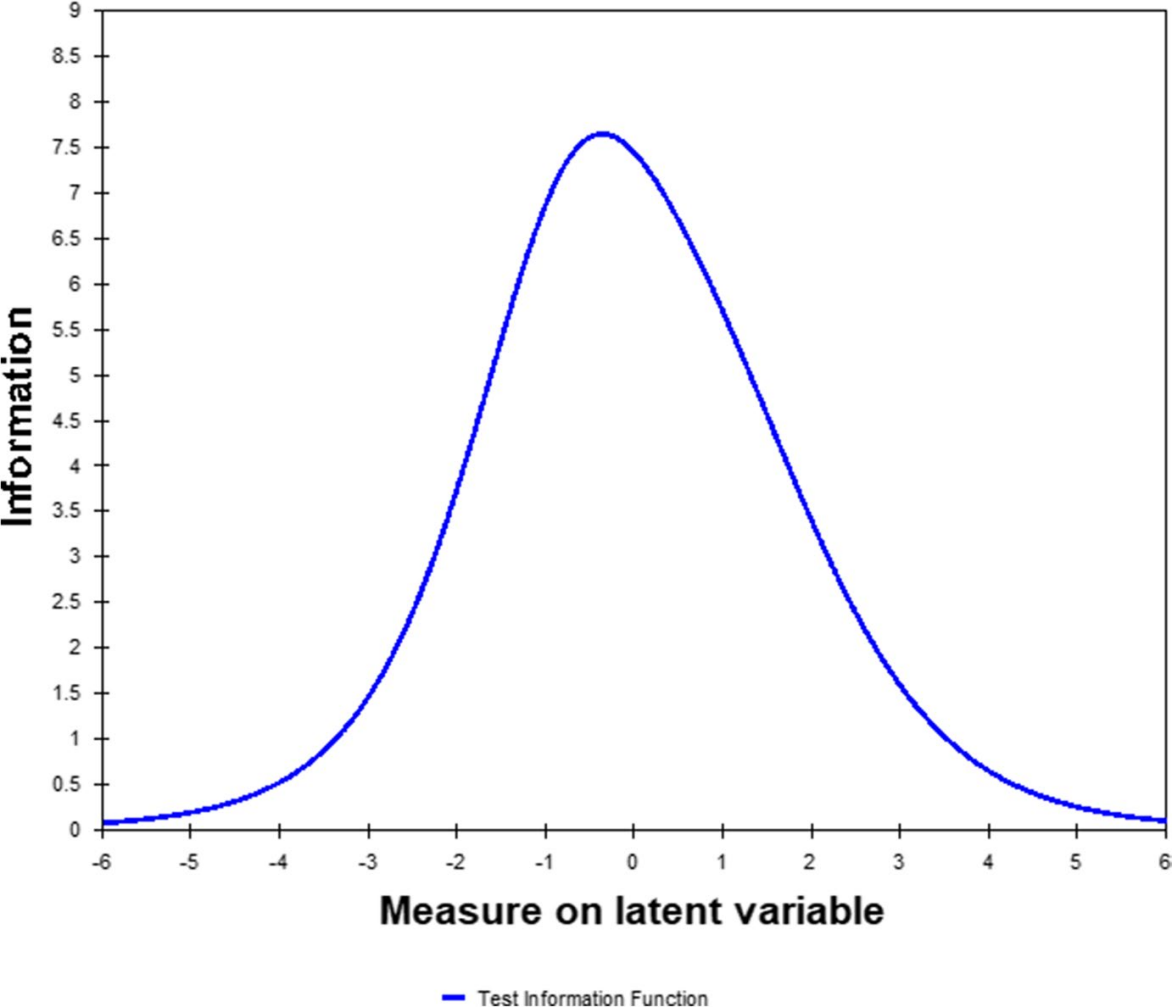
Test information function in Rasch analysis

The plot of the test information function in Rasch for the 8-item version of the PIS is reported in Fig. 2. The plot reflects that the scale as a whole demonstrated an information peak when the latent variable ranges between − 1.5 and 1.5. Thus, it confirms that levels of the latent variable between − 1.5 and 1.5 are assessed with some precision.

## Discussion

South Africa’s history is characterized by segregationist policies and differentiated education for different racial groups. This had a significant bearing on the teaching profession and teacher professional identity (Davids, 2018). Under the apartheid system of governance, teaching was one of the few occupations with high status and good working conditions for black people. However, it remained highly racialized and gendered (Cross & Ndofirepi, 2015) — there were few opportunities for advancement among black people and women were often inhibited from entering professional environments due to patriarchal gender roles. Although there have been significant advances in education policy and attempts to promote delivery and access to quality education, the teaching profession is undervalued in the country (Davids, 2018). Low pay, poor working conditions in government funded schools, and limited opportunities for advancement have impacted on entry into the profession. Efforts at racial integration have not been wholly successful as teachers are often ill-equipped to challenge their own ingrained prejudices as well as those of students and parents (Cross & Ndofirepi, 2015). Furthermore, the significant discrepancies in access to resources between schools in more privileged settings compared to those in rural areas have meant that few teachers are willing to work in rural communities (Du Plessis & Mestry, 2019). Those that remain in underprivileged areas do so from a sense of moral obligation to the community (Du Plessis & Mestry, 2019).

Cross & Ndofirepi (2015) argue that teacher identity in South Africa has been shaped by the socio-cultural history of the country, personal values, pedagogical practices, and government policies. Teacher identity is intertwined with the ways in which teachers teach and with the relationships they build with their students and peers. The enactment of teacher identity impacts the learning experience of students and the quality of education provided (Zembylas & Chubbuck, 2018). Most research on teacher identity has been either theoretical or qualitative and there remains limited quantitative research that has assessed the rigor of instruments aimed at assessing teacher identity (Hanna et al., 2019; Lamote & Engels, 2010; Zembylas & Chubbuck, 2018). This type of research is important for various reasons including the provision of more individualized support for teachers at various stages of identity formation, the testing and advancement of developmental models of teacher identity formation and the assessment of patterns in teacher identity formation in relation to various independent variables (Hanna et al., 2019).

It is against this backdrop that the current study examined the psychometric properties of the PIS using a parametric item response theory (Rasch analysis), a nonparametric item response theory (Mokken analysis), and classical test theory. The three approaches confirmed the reliability and validity of a reduced 8-item version of the PIS. First, the scale demonstrated satisfactory internal consistency (Cronbach’s alpha and Mokken’s R_ho_). Two subscales identified through EFA and CFA also had acceptable reliability coefficients.

Second, strong evidence for the validity of the 8-item scale was identified. The good model-fit indices supporting the conceptualization of the 8-item PIS, the contribution of all items to the measurement of professional identification (item-total correlations > 0.50), and the sensitivity of the scale to distinguish between different levels of performers (person separation reliability 0.67–80 is fair, monotonicity not violated) all support good construct validity for the PIS. Additionally, there was no violation of IIO, which indicates that respondents with the same level of the latent construct responded consistently across items. The existence of an item difficulty hierarchy (item separation index > 3, item separation reliability > 0.80), good Rasch measures of model fit (infit and outfit MnSq between 0.5–1.5), and measurement invariance across groups (DIF < 0.50) all further support good construct validity for the PIS.

Further, all factor loadings were significant, and AVE was > 0.50 and below CR, which demonstrates that items have a high proportion of variance in common. This finding provides evidence of convergent validity. Professional identity as a latent construct accounted for more of the variance in the items than the variance it shared with teaching satisfaction and life satisfaction (AVE > MSV and ASV), which provides support for discriminant validity. Professional identification was positively correlated with teaching satisfaction and life satisfaction. The two extracted subscales, professional pride and professional discontent, were also correlated with teaching satisfaction and life satisfaction. These relationships provide evidence of concurrent validity.

Third, while the model fit indices in CFA support a one-factor 8-item scale, they also showed comparable fit statistics for a bifactor model consisting of a total scale and two subscales. AISP (Mokken analysis), a PCA of residuals (Rasch analysis), and ancillary bifactor indices also demonstrated that the PIS should be regarded as multidimensional. The ancillary bifactor indices indicated that professional pride and professional discontent account for a sufficient amount of variance above that accounted for by professional identity as a total scale. Brown and colleagues (1986) extracted three factors, with all the positive items loading on one factor and the negative items on two other factors. However, they elected to use a single-scale score only. The current results demonstrate that, in addition to the total PIS scale, there are two meaningful and reliable subscales. In sum, CTT, Rasch analysis, and Mokken analysis demonstrate that the 8-item PIS is a reliable, valid, and multidimensional measure of professional identification with a total scale and two subscales of professional pride and professional discontent. This 8-item scale is found to be valid and reliable in the South African context.

## Limitations

The existing literature has identified many processes and traits that comprise professional identity. The current study identified two subscales of PIS—professional pride and professional discontent—in the South African context. Additional qualitative studies should be conducted to further explore the daily experiences of pride and discontent in South Africa. The study should be considered within the context of its limitations. Although efforts were made to access a representative sample of South Africa, most teachers in the study sample resided in the Western Cape. A replication of this study on a larger scale is recommended, which could include representation of teachers from other provinces of the country. A significant contribution of the study is that two main approaches, classical test theory and item response theory, were used in the analysis. Measurement validation in cross-cultural research is an ongoing process; thus, future studies should attempt to access a more representative sample of teachers in South Africa according to province, age, gender, and rural and urban areas.

## Conclusion

CTT, Rasch, and Mokken analyses were used to validate the PIS among a sample of teachers. The analysis confirmed that an 8-item scale with two subscales—professional pride and professional discontent—is a valid and reliable measure of teachers’ identification with the teaching profession. Future studies in different cultural settings would be needed to further validate our findings.

## Data Availability

The datasets generated during and/or analyzed during the current study are available from the corresponding author on reasonable request.
